# Impact of COVID‐19, gender, race, specialty and seniority on mental health during surgical training: an international study

**DOI:** 10.1111/ans.17980

**Published:** 2022-08-18

**Authors:** Joshua G. Kovoor, Georgia R. Layton, Joshua R. Burke, James A. Churchill, Jonathan Henry W. Jacobsen, Jessica L. Reid, Suzanne Edwards, Eyad Issa, Tamsin J. Garrod, Julian Archer, David R. Tivey, Wendy J. Babidge, Ashley R. Dennison, Guy J. Maddern

**Affiliations:** ^1^ Discipline of Surgery, The Queen Elizabeth Hospital University of Adelaide Adelaide South Australia Australia; ^2^ Royal Australasian College of Surgeons Australia; ^3^ University Hospitals of Leicester NHS Trust Leicester UK; ^4^ Department of Gastrointestinal Surgery Leeds Teaching Hospitals, NHS Trust UK; ^5^ St George Hospital Sydney New South Wales Australia; ^6^ Adelaide Health Technology Assessment, School of Public Health University of Adelaide Adelaide South Australia Australia; ^7^ Faculty of Medicine, Nursing and Health Sciences Monash University Victoria Australia

**Keywords:** COVID‐19, gender, mental health, race, surgical training

## Abstract

**Background:**

Superior patient outcomes rely on surgical training being optimized. Accordingly, we conducted an international, prospective, cross‐sectional study determining relative impacts of COVID‐19, gender, race, specialty and seniority on mental health of surgical trainees.

**Method:**

Trainees across Australia, New Zealand and UK enrolled in surgical training accredited by the Royal Australasian College of Surgeons or Royal College of Surgeons were included. Outcomes included the short version of the Perceived Stress Scale, Oxford Happiness Questionnaire short scale, Patient Health Questionnaire‐2 and the effect on individual stress levels of training experiences affected by COVID‐19. Predictors included trainee characteristics and local COVID‐19 prevalence. Multivariable linear regression analyses were conducted to assess association between outcomes and predictors.

**Results:**

Two hundred and five surgical trainees were included. Increased stress was associated with number of COVID‐19 patients treated (*P* = 0.0127), female gender (*P* = 0.0293), minority race (*P* = 0.0012), less seniority (*P* = 0.001), and greater COVID‐19 prevalence (*P* = 0.0122). Lower happiness was associated with training country (*P* = 0.0026), minority race (*P* = 0.0258) and more seniority (*P* < 0.0001). Greater depression was associated with more seniority (*P* < 0.0001). Greater COVID‐19 prevalence was associated with greater reported loss of training opportunities (*P* = 0.0038), poor working conditions (*P* = 0.0079), personal protective equipment availability (*P* = 0.0008), relocation to areas of little experience (*P* < 0.0001), difficulties with career progression (*P* = 0.0172), loss of supervision (*P* = 0.0211), difficulties with pay (*P* = 0.0034), and difficulties with leave (*P* = 0.0002).

**Conclusion:**

This is the first study to specifically describe the relative impacts of COVID‐19 community prevalence, gender, race, surgical specialty and level of seniority on stress, happiness and depression of surgical trainees on an international scale.

## Graphical abstract

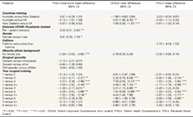

## Introduction

COVID‐19 has affected surgical systems and the staff within them.^1^ The lives and experiences of residents undergoing surgical training have been disrupted.^2^ Resident operative volume has reduced, potentially affecting technical skill development.^3^ Pandemic changes to surgical research^4^ may have inhibited the formation of academic fundamentals. Reorganization of rotations and the delivery of surgical care^5^, ^6^ may have impacted the surgical training experience globally.

Mental health has been a prominent issue throughout the COVID‐19 pandemic,^7^ particularly for healthcare workers.^8^ Although surgical resident grit is generally high,^9^ the mental load associated with surgical training can potentially create high levels of burnout, stress, and distress, and depression.^10^, ^11^ These impacts could be more significant in settings of higher COVID‐19 caseload, where surgical staff may be under greater stress.^12^ Similarly, female sex and visible minority status are associated with poor surgical trainee experiences.^13^ Gender discrimination, sexual harassment, and other forms of mistreatment are frequently reported by women in surgical training, and this is associated with increased burnout and suicidality.^14^, ^15^ Racial discrimination is experienced by a considerable proportion of non‐white surgical trainees, resulting in compounded psychological distress.^16^

Producing the best outcomes for surgical patients relies on the surgical training experience being optimized. This is especially important in the setting of COVID‐19. Therefore, to inform surgery during the pandemic, we conducted a cross‐sectional study across Australia, New Zealand and the UK aiming to determine the relative impacts of COVID‐19, gender, race, specialty and seniority on mental health of residents undergoing surgical training.

## Methods

### Study design and oversight

This prospective, cross‐sectional study was undertaken in collaboration with the Royal Australasian College of Surgeons (RACS), the Association of Surgeons in Training (ASiT), and the Royal Australasian College of Surgeons Trainees' Association (RACSTA). It followed the STROBE guidelines for reporting observational studies.^17^ It was conducted from 19 April 2021 to 3 September 2021 across Australia, New Zealand and the UK. Prior to commencement, the initial study protocol was reviewed by the Board of Surgical Education and Training of RACS, who provided formal endorsement of the study and research questions. Members of executive bodies within RACS, ASiT and RACSTA had a role in the design and conduct of the study as co‐authors and collaborators, including data acquisition, analysis and interpretation, revising the article critically for important intellectual content, and final decision to submit the manuscript for publication. Ethical approval was obtained from the Central Adelaide Local Health Network Human Research Ethics Committee (reference number: 14333).

### Participants and data collection

The study population comprised residents across Australia, New Zealand and the UK enrolled in a formal surgical training program accredited by the RACS or the Royal College of Surgeons (RCS). All years of training and surgical sub‐specialties were included. An electronic, anonymised questionnaire was developed using a web application (SurveyMonkey, San Mateo, CA, USA) and distributed to eligible surgical trainees using email and social media. Emails including the survey link were distributed by the RACS, and the survey link was posted on ASiT social media accounts.

### Measures

The primary outcome was the four‐item short version of the Perceived Stress Scale (PSS‐4).^18^ Other outcomes of interest included the Oxford Happiness Questionnaire short scale (OHQ‐8),^19^ the Patient Health Questionnaire‐2 (PHQ‐2),^20^ proportion of expected pre‐pandemic operating volume, and non‐validated measures of surgical training experience during the COVID‐19 pandemic (Data S1). The relationship between these outcomes and various predictors, including resident characteristics and local COVID‐19 prevalence, was investigated. Local COVID‐19 prevalence was calculated by extracting data from the COVID‐19 Data Repository by the Center for Systems Science and Engineering (CSSE) at Johns Hopkins University.^21^ The date of the trainees' survey responses was used to extract daily COVID‐19 case rates at the state level if trainees were in Australia and New Zealand or country level if they were in the UK. Details on measurement of outcomes, including survey scores and raw data, are available on reasonable request to the corresponding author.

### Statistical analysis

All statistical analysis was performed using SAS 9.4 (SAS Institute Inc., Cary, NC, USA). Multivariable linear regressions were conducted to assess the association between outcomes: PSS‐4,^18^ OHQ‐8,^19^ PHQ‐2,^20^ or proportion of expected pre‐pandemic operative activity, and predictors: COVID‐19 survey questions (supplement), and demographic and clinical variables (gender, race/ethnicity, surgical specialty, country of training, year of training, estimated COVID‐19 patients treated and 7 day average daily COVID‐19 case rates). Assumptions of a linear model were assessed by inspection of scatter plots (testing homoscedasticity) and histograms (assessing normality of residuals). Goodness of fit and model selection were evaluated using Pearson residuals and by consideration of AIC, AOIC and BIC, respectively. To infer the impact of each covariate, backwards elimination was performed, removing the predictor with the highest *P*‐value until all *P*‐values were less than 0.2.^22^ Country of training was retained as an *a priori* predictor in all models. Results were presented as mean or mean difference with 95% confidence intervals and were considered statistically significant if the *P*‐value was ≤0.05.

## Results

### Study sample

A total of 205 residents undergoing surgical training accepted the invite to participate in the study, 146 (71.2%) of whom were primarily based in Australia, 34 (16.6%) in New Zealand, and 25 (12.2%) in the UK. Regarding gender, 94 (45.9%) residents reported being female, 110 (53.7%) male and 1 (0.5%) non‐binary. Regarding race/ethnicity, 53 (25.9%) residents reported being of minority ethnic background. Regarding surgical specialty, 108 (52.7%) were undertaking primarily training in general surgery, 34 (16.6%) orthopaedic surgery, 12 (5.9%) plastic and reconstructive surgery, 12 (5.9%) otolaryngology, head and neck surgery, 10 (4.9%) paediatric surgery, 9 (4.4%) urology, 8 (3.9%) neurosurgery, 7 (3.4%) cardiothoracic surgery, 3 (1.5%) vascular surgery and 2 (1.0%) oral and maxillofacial surgery. Median year of surgical training was 2 (IQR: 1–4). Median proportion of expected operating volume completed was 79% (IQR: 60–90%). Median estimated number of COVID‐19 patients treated was 0 (IQR: 0–3; range: 0–216). 6 (2.9%) residents had a past diagnosis of COVID‐19, and 17 (8.3%) had a family member with a past diagnosis of COVID‐19. 18 (8.8%) residents had travelled from overseas to undertake training and 16 (7.8%) were undertaking training in less than full time fashion.

### Stress

Statistically significant associations were found between PSS‐4 score and estimated number of COVID‐19 patients treated (*P* = 0.0127), gender (*P* = 0.0293), minority ethnic background (*P* = 0.0012), and year of surgical training (*P* = 0.001), while adjusting for all other covariates in the model. There was also a statistically significant association between PSS‐4 score and reported loss of training opportunities (*P* = 0.0012), and reported difficulties with exams, competence assessments and interviews (*P* = 0.0026) (Table 1). There was a statistically significant association between PSS‐4 score and seven‐day average daily COVID‐19 case rates at the resident's location of surgical training (*P* = 0.0122; Table 2).

**Table 1:** Multivariable linear regression of OHQ‐8, PHQ‐2, PSS‐4 score versus demographics

Predictor	PSS‐4 total score mean difference (95% CI)	OHQ‐8 mean difference (95% CI)	PHQ‐2 mean difference (95% CI)
***Countries training***			
Australia versus New Zealand	0.81 (−0.29, 1.91)	−1.99 (−4.83, 0.85)	0.23 (−0.34, 0.81)
Australia versus UK	−0.16 (−1.81, 1.49)	5.01 (1.66, 8.35) **	−0.44 (−1.13, 0.25)
New Zealand versus UK	−0.97 (−2.86, 0.92)	7.00 (2.93, 11.07) ***	−0.67 (−1.50, 0.16)
***Estimate COVID‐19 patients treated***			
Per 1 patient increase	0.03 (0.01, 0.05) **		
***Gender***			
Female versus male	0.91 (0.09, 1.74) *		
***Fulltime***			
Fulltime versus less than fulltime			0.72 (−0.08, 1.53)
***Minority ethnic background***			
No versus yes	−1.58 (−2.53, −0.62) ***	2.76 (0.33, 5.20)	−0.35 (−0.85, 0.15)
***Surgical specialty***			
General versus orthopaedic	−1.10 (−2.27, 0.07)		
General versus other	−0.45 (−1.36, 0.45)		
Orthopaedic versus oOther	0.64 (−0.62, 1.90)		
***Year surgical training***			
1 versus 2	−0.14 (−1.32, 1.04)	0.01 (−2.97, 2.99)	−0.31 (−0.92, 0.31)
1 versus 3	−1.52 (−2.77, −0.27) **	5.32 (2.15, 8.49) ***	−0.80 (−1.45, −0.14) *
1 versus 4	−2.22 (−3.48, −0.96) ***	7.25 (4.03, 10.47) ***	−1.60 (−2.26, −0.94) ***
1 versus 5+	−1.64 (−2.82, −0.46) ***	4.46 (1.49, 7.43) **	−1.17 (−1.78, −0.56) ***
2 versus 3	−1.38 (−2.77, 0.01) *	5.31 (1.73, 8.89) **	−0.49 (−1.22, 0.25)
2 versus 4	−2.08 (−3.51, −0.66) **	7.24 (3.59, 10.89) ***	−1.29 (−2.04, −0.54) ***
2 versus 5+	−1.50 (−2.82, −0.18) *	4.44 (1.04, 7.85)	−0.87 (−1.56, −0.17) *
3 versus 4	−0.70 (−2.16, 0.76)	1.93 (−1.83, 5.70)	−0.80 (−1.58, −0.03) *
3 versus 5+	−0.12 (−1.50, 1.27)	−0.86 (−4.41, 2.69)	−0.38 (−1.11, 0.35)
4 versus 5+	0.58 (−0.83, 1.99)	−2.80 (−6.37, 0.78)	0.43 (−0.30, 1.16)

*P < 0.05, **P < 0.01, ***P < 0.001. OHQ‐8, Oxford Happiness Questionnaire short scale‐8; PHQ‐2, Patient Health Questionnaire‐2; PSS‐4, Perceived Stress Scale‐4.

**Table 2:** Multivariable linear regression of OHQ‐8, PHQ‐2, PSS‐4 score versus demographics and survey questions investigating the effect on individual stress levels of various training experiences affected by COVID‐19

Predictor	PSS‐4 Total score mean difference (95% CI)	OHQ‐8 mean difference (95% CI)	PHQ‐2 mean difference (95% CI)
***COVID survey questions***			
Loss training opportunities	0.03 (0.01, 0.05) **	−0.04 (−0.09, 0.01)	0.09 (−0.01, 0.19)
Poor working conditions	0.01 (−0.00, 0.03)	−0.06 (−0.11, −0.02) **	0.08 (−0.01, 0.16)
Personal protective equipment	−0.01 (−0.03, 0.00)	0.07 (0.03, 0.11) ***	−0.13 (−0.22, −0.04) **
Difficulties exams assessments	0.02 (0.01, 0.03) **	−0.03 (−0.06, 0.01)	0.07 (−0.00, 0.14)
Difficulties with pay and contracts		−0.04 (−0.09, −0.00) *	0.11 (0.03, 0.20) *
Difficulties obtaining leave	0.01 (−0.00, 0.03)	−0.03 (−0.07, 0.00)	0.07 (−0.01, 0.15)
***Seven day average daily COVID‐19 case rates at location***			
Per 1000 increase	0.0946 (0.0206, 0.1685) **	−0.1876 (−0.3848, 0.0095)	0.0288 (−0.0092, 0.0668)
***Countries training***			
Australia versus New Zealand	0.32 (−0.65, 1.29)	−0.31 (−3.00, 2.38)	0.02 (−0.53, 0.57)
Australia versus UK	1.08 (−0.38, 2.54)	1.75 (−1.33, 4.83)	0.27 (−0.36, 0.90)
New Zealand versus UK	0.76 (−0.95, 2.48)	2.06 (−1.87, 5.99)	0.25 (−0.56, 1.05)
***Estimate COVID‐19 patients treated***			
Per 1 patient increase	0.02 (0.00, 0.04)		
***Minority ethnic background***			
No versus yes	−1.14 (−1.98, −0.30)		
***Year surgical training***			
1 versus 2	−0.28 (−1.33, 0.77)	0.96 (−1.92, 3.85)	−0.60 (−1.19, −0.01) *
1 versus 3	−1.57 (−2.66, −0.48) **	5.92 (2.97, 8.88) ***	−1.01 (−1.62, −0.41) **
1 versus 4	−0.78 (−1.91, 0.34)	3.99 (0.93, 7.05) **	−1.04 (−1.66, −0.41) **
1 versus 5+	−0.50 (−1.53, 0.54)	2.70 (−0.12, 5.51)	−0.69 (−1.27, −0.12) *
2 versus 3	−1.29 (−2.52, −0.06) *	4.96 (1.62, 8.30) **	−0.42 (−1.10, 0.27)
2 versus 4	−0.51 (−1.82, 0.80)	3.03 (−0.59, 6.64)	−0.44 (−1.18, 0.30)
2 versus 5+	−0.22 (−1.43, 0.99)	1.73 (−1.58, 5.04)	−0.10 (−0.78, 0.58)
3 versus 4	0.79 (−0.52, 2.09)	−1.93 (−5.49, 1.62)	−0.02 (−0.75, 0.71)
3 versus 5+	1.07 (−0.14, 2.28)	−3.23 (−6.52, 0.06)	0.32 (−0.36, 0.99)
4 versus 5+	0.29 (−0.90, 1.47)	−1.29 (−4.50, 1.92)	0.34 (−0.32, 1.00)

*P < 0.05, **P < 0.01, ***P < 0.001. OHQ‐8, Oxford Happiness Questionnaire short scale‐8; PHQ‐2, Patient Health Questionnaire‐2; PSS‐4, Perceived Stress Scale‐4.

### Happiness

Statistically significant associations were found between OHQ‐8 score and country of surgical training (*P* = 0.0026), minority ethnic background (*P* = 0.0258) and year within the surgical training program (*P* < 0.0001) while adjusting for all other covariates in the model (Table 1). Residents in Australia had a mean OHQ‐8 score 5.01 units greater than those in the UK (*P* = 0.0033). Residents in New Zealand had a mean OHQ‐8 score 7 units greater than those in the UK (*P* = 0.0007). There was no statistically significant difference in mean OHQ‐8 scores between residents in Australia and those in New Zealand. There was also a statistically significant association between OHQ‐8 score and reported poor working conditions (*P* = 0.0047), reported issues with personal protective equipment (*P* = 0.0014), and reported difficulties with pay and contracts (*P* = 0.0343) (Table 2). There was no statistically significant association between OHQ‐8 score and seven‐day average daily COVID‐19 case rates at the resident's location of surgical training (*P* = 0.0622; Table 2).

### Depression

A statistically significant association was found between PHQ‐2 score and year within the surgical training program (*P* < 0.0001), while adjusting for other covariates in the model, such that more senior trainees more likely to record scores indicating depression (Table 1). There was also a statistically significant association between PHQ‐2 score and reported issues with personal protective equipment (*P* = 0.0031), and reported difficulties with pay and contracts (*P* = 0.0094) (Table 2). There was no statistically significant association between PHQ‐2 score and seven‐day average daily COVID‐19 case rates at the resident's location of surgical training (*P* = 0.1378; Table 2).

### Operating volume

Statistically significant associations were found between proportion of expected operating volume completed and country of surgical training (*P* = 0.0142), estimated number of COVID‐19 patients treated (*P* = 0.0301), and minority ethnic background (*P* = 0.0172), adjusting for all other covariates in the model (Table 3). Residents in Australia had a mean proportion of expected operating volume completed that was 15.75% greater than those in the UK (*P* = 0.0095). Residents in New Zealand had a mean proportion of expected operating volume completed that was 19.94% greater than those in the UK (*P* = 0.0039). There was no statistically significant difference between mean proportion of expected operating volume completed between residents in Australia versus those in New Zealand. There was a statistically significant association between proportion of expected operating volume completed and reported loss of training opportunities (*P* = 0.0058) (Table 4). There was a statistically significant association between proportion of expected operating volume completed and seven‐day average daily COVID‐19 case rates at the resident's location of surgical training (*P* < 0.0001; supplement).

**Table 3:** Multivariable linear regression of proportion expected operating activity versus demographics

Predictor	Comparison	Mean difference (95% CI)	Comparison *P*‐value	Global *P*‐value
Countries training	Australia versus New Zealand	−4.19 (−12.04, 3.67)	0.2960	0.0142
	Australia versus UK	15.75 (3.85, 27.64)	0.0095	
	New Zealand versus UK	19.94 (6.40, 33.47)	0.0039	
Estimate patients COVID treated	Per 10 patient increase	−1.69 (−3.22, −0.16)		0.0301
Minority ethnic background	No versus yes	8.39 (1.49, 15.30)		0.0172
Surgical specialty	General versus orthopaedic	5.45 (−2.68, 13.58)	0.1889	0.1751
	General versus other	5.56 (−0.99, 12.11)	0.0964	
	Orthopaedic versus other	0.11 (−8.65, 8.87)	0.9806	

**Table 4:** Linear regression of proportion expected operating activity versus demographics and survey questions investigating the effect on individual stress levels of various training experiences affected by COVID‐19

Predictor	Comparison	Mean difference (95% CI)	Comparison *P*‐value	Global *P*‐value
Loss training opportunities	Per 10 unit increase	−1.91 (−3.26, −0.55)		0.0058
Countries training	Australia versus New Zealand	−2.85 (−10.21, 4.51)	0.4482	0.1232
	Australia versus UK	10.00 (−0.81, 20.80)	0.0697	
	New Zealand versus UK	12.85 (0.41, 25.29)	0.0430	
Difficulties regarding career	Per 10 unit increase	−0.87 (−1.91, 0.17)		0.1005
Estimate patients with COVID‐19 treated	Per 10 patient increase	−1.10 (−2.49, 0.30)		0.1248
Loss of supervision	Per 10 unit increase	−0.89 (−2.02, 0.24)		0.1231
Minority ethnic background	No versus yes	6.92 (0.55, 13.29)		0.0332

### Surgical training during COVID‐19

The survey questions investigated the effect on individual stress levels of various training experiences affected by COVID‐19. Reported loss of training opportunities was significantly associated with country of surgical training (*P* = 0.0005), gender (*P* = 0.0025) and year of surgical training (*P* = 0.0003), while adjusting for other covariates in the model. Reported poor working conditions were significantly associated with estimated number of COVID‐19 patients treated (*P* = 0.0216), gender (*P* = 0.0168), minority ethnic background (*P* = 0.0267) and year of surgical training (*P* = 0.0231). Increased reported issues with personal protective equipment availability were significantly associated with gender (*P* < 0.0001) and seven‐day average daily COVID‐19 case rates at the resident's location of surgical training (*P* = 0.0467). Increased reported relocation to areas of little experience was significantly associated with the resident or family member being diagnosed with COVID‐19 (*P* = 0.049), and minority ethnic background (*P* = 0.0311). Reported difficulties in career progression were significantly associated with country (*P* = 0.0088) and year (*P* < 0.0001) of surgical training. Reported loss of supervision and mentoring was significantly associated with country of surgical training (*P* = 0.0382), estimated number of COVID‐19 patients treated (*P* = 0.0248) and gender (*P* = 0.0014). Reported difficulties with examinations and assessments were significantly associated with country of surgical training (*P* = 0.032), gender (*P* = 0.0157), and year of surgical training (*P* < 0.0001). Reported difficulties with pay and contracts were significantly associated with country of surgical training (*P* < 0.0001), self or family diagnosis of COVID‐19 (*P* = 0.0073), having undertaken overseas travel for training (*P* = 0.0452), and year of surgical training (*P* = 0.0014). Reported difficulties obtaining leave were significantly associated with country of surgical training (*P* < 0.0001) and gender (*P* = 0.0089).

Greater 7‐day average daily COVID‐19 case rates at the resident's location of surgical training were significantly associated with greater reported loss of training opportunities (*P* = 0.0038), reported poor working conditions (*P* = 0.0079), personal protective equipment availability (*P* = 0.0008), reported relocation to areas of little experience (*P* < 0.0001), reported difficulties with career progression (*P* = 0.0172), reported loss of supervision or mentoring (*P* = 0.0211), reported difficulties with pay or contracts (*P* = 0.0034), and reported difficulties with obtaining leave (*P* = 0.0002) (Table 5).

**Table 5:** Univariate linear regression of survey questions investigating the effect on individual stress levels of various training experiences affected by COVID‐19 versus 7 day average daily COVID‐19 rate at location

Outcome	Predictor	Comparison	Mean estimate (95% CI)	Global *P*‐value
Loss of training opportunities	Seven day average daily COVID‐19 rate at location	Per 1000 increase	1.01 (0.33, 1.69)	0.0038
Poor working conditions	Seven day average daily COVID‐19 rate at location	Per 1000 increase	0.99 (0.26, 1.73)	0.0079
Personal protective equipment issues	Seven day average daily COVID‐19 rate at location	Per 1000 increase	1.18 (0.49, 1.87)	0.0008
Relocation to areas of little expertise	Seven day average daily COVID‐19 rate at location	Per 1000 increase	1.38 (0.70, 2.05)	<0.0001
Difficulties regarding career progression	Seven day average daily COVID‐19 rate at location	Per 1000 increase	1.01 (0.18, 1.83)	0.0172
Loss of supervision or mentoring	Seven day average daily COVID‐19 rate at location	Per 1000 increase	0.87 (0.13, 1.61)	0.0211
Difficulties with examinations or assessments	Seven day average daily COVID‐19 rate at location	Per 1000 increase	0.68 (−0.20, 1.55)	0.1283
Difficulties with pay or contracts	Seven day average daily COVID‐19 rate at location	Per 1000 increase	1.03 (0.34, 1.72)	0.0034
Difficulties obtaining leave	Seven day average daily COVID‐19 rate at location	Per 1000 increase	1.46 (0.70, 2.23)	0.0002

## Discussion

This is the first study to specifically describe the relative impacts of COVID‐19 community prevalence, gender, race, surgical specialty and level of seniority on stress, happiness and depression of residents undergoing surgical training on an international scale. This study adds to the literature on the impact of COVID‐19 on surgical training^23^ by considering the issue in an Australian and New Zealand context, comparing this to a UK context, and considering the impact of COVID‐19 in combination with the demographic characteristics of the surgical trainees. Minorities of gender and race/ethnicity were adequately represented within the study sample, particularly in comparison to published datasets characterizing rates of representation in surgical training.^24^ Greater seven‐day average daily COVID‐19 case rates at the resident's location of surgical training were associated with increased resident stress, lower proportion of expected operating volume completed and numerous challenges in resident experience during the pandemic. Greater resident stress was associated with greater number of COVID‐19 patients treated, female gender, minority ethnic background and generally more junior year within surgical training. Lower resident happiness was associated with country of surgical training, with residents in UK having significantly less happiness on average than those in Australia or New Zealand, being of a minority ethnic background and generally more senior year in surgical training. Greater resident depression was associated with being a more senior year in surgical training. Residents in Australia and New Zealand had completed a significantly greater mean proportion of their expected operating volumes. No significant differences in mental health outcomes were found between residents undergoing training in different surgical specialties.

The mental health of residents undergoing surgical training has become explored in greater detail within the literature of recent years.^25^ The impact that COVID‐19 has had on surgical training is summarized in a systematic review by Hope *et al*.^23^ Women leave surgical training at greater rates than men, and can be helped via interventions that alert the possibility of unplanned negative effects during training without focusing on gender.^26^ These findings are reflected in these analyses, demonstrating that female residents are experiencing significantly greater stress during the COVID‐19 pandemic. Similarly, surgical trainees in racial minority groups are largely underrepresented across specialties and many experience racial/ethnic discrimination leading to significant distress.^16^ This study confirmed that mental health for residents of minority ethnic backgrounds was significantly poorer during COVID‐19. This study adds pertinent, novel data to the literature in the finding that residents at more senior stages of their surgical training experience significantly greater levels of stress, lower levels of happiness, and greater levels of depression relative to those more junior. However, this may be a product of greater responsibility being suddenly taken on by senior residents due to COVID‐19. Further, other causes of senior trainee stress apart from COVID‐19 may have influenced the present results, particularly considering the low rate of COVID‐19 patients treated by the study population.

A decline in overall surgical resident operative autonomy has been reported over recent decades, and this may have been augmented by COVID‐19.^3^ This study found that increased community prevalence of COVID‐19 was significantly associated with a decreased proportion of expected operating volume being completed. This was agreed with the finding that on average, residents in both Australia and New Zealand had significantly greater completion of their experienced operating volume during the pandemic, and reported significantly fewer losses of training opportunities, than those in the UK. Female gender and greater seniority were the other factors with significantly increased reporting of loss of training opportunities during COVID‐19. Similarly, this study identified resident factors significantly associated with increased difficulties with poor working conditions, personal protective equipment availability, relocation, career progression, supervision, pay and leave during the COVID‐19 pandemic. Given the prominent role that it plays on resident mental health, decreasing community COVID‐19 caseload should be a priority for surgical system management and staff. To facilitate this, appropriate screening and testing for COVID‐19 in surgical patients should be implemented,^27^ and vaccination should be delivered at a population level.

This study has multiple limitations. There was a relatively low sample size, particularly of residents undergoing training in the UK, however despite this, statistically significant associations between resident factors and mental health were able to be identified. Potential bias may have been incurred as the residents that responded to the email invitation to participate may have been those with poorer experiences during the pandemic, however, sufficient breadth in responses were received to produce significant differences within analyses of the study outcomes. Further, because this method of surveying was undertaken, only participants that responded to the invitation were recorded, and an overall rate of potential participants that did not partake in the survey could not be calculated. As the primary outcome of stress was self‐reported, there is the potential either under or over‐reporting based on participant characteristics, including gender. For the associations between the survey questions and other outcomes that were investigated, only statistically significant associations were able to be reported, and insufficient data were present to infer causality. Accordingly, these results have also not been discussed in detail within the Discussion. The low rate of patients treated was dependent on the time that the survey was conducted, and may add bias to the results in the ongoing milieu of COVID‐19. The proportion of General Surgery residents within the cohort that responded was higher than that real‐life proportion, potentially adding bias. Although residents were included from multiple countries within the study cohort, the translatability of findings to countries other than those included, particularly those of middle and low income, may be limited.

## Conclusions

This international study identifies important factors that impact resident mental health during surgical training and is relevant both during and after the COVID‐19 pandemic. To improve patient outcomes and surgical training experience for future waves of COVID‐19, surgical colleges and training associations around the world should action these identified associations. This may include providing methods for the protection of minorities, the promotion of gender equality and the development of new models of training to compensate for the dilution in training during the COVID‐19 pandemic.

## Author contributions

**Joshua G. Kovoor:** Conceptualization; investigation; writing – original draft; writing – review and editing. **Georgia R. Layton:** Conceptualization; investigation; writing – review and editing. **Joshua R. Burke:** Conceptualization; investigation; writing – review and editing. **James A. Churchill:** Conceptualization; investigation; writing – review and editing. **Jonathan Henry W. Jacobsen:** Conceptualization; investigation; writing – review and editing. **Jessica L. Reid:** Conceptualization; investigation; writing – review and editing. **Suzanne Edwards:** Conceptualization; investigation; writing – review and editing. **Eyad Issa:** Conceptualization; investigation; writing – review and editing. **Tamsin J. Garrod:** Conceptualization; investigation; writing – review and editing. **Julian Archer:** Conceptualization; investigation; writing – review and editing. **David R. Tivey:** Conceptualization; investigation; writing – review and editing. **Wendy J. Babidge:** Conceptualization; investigation; writing – review and editing. **Ashley Dennison:** Conceptualization; investigation; writing – review and editing. **Guy J. Maddern:** Conceptualization; investigation; supervision; writing – review and editing.

## Disclosure statement

One of the authors is an Editorial Board member of the journal and co‐author of this article. They were excluded from the peer‐review process and all editorial decisions related to the acceptance and publication of this article. Peer‐review was handled independently by members of the Editorial Board to minimize bias.

## Conflict of interest

None declared.

## Supporting information


**Appendix S1.** Supporting Information
