# Supplementary material for: Impact of COVID‐19, gender, race, specialty and seniority on mental health during surgical training: an international study
Source: ANZ J Surg. 2022 Aug 18;92(9):2094–101. doi: 10.1111/ans.17980 (PMC9538543; doi:10.1111/ans.17980)
Supplement: Supplementary file 1 — Appendix S1. Supporting Information [file ANS-92-2094-s001.docx]

**APPENDIX**

**SURVEY QUESTIONS**

1. What is your **gender**?
   1. Female
   2. Male
   3. Non-binary
   4. Prefer not to say
2. What **countries** do you undertake your surgical training in? Select all that apply.
   1. Australia
   2. New Zealand
   3. England
   4. Scotland
   5. Wales
   6. Northern Ireland
3. What **hospitals** have you worked in since 11 March 2020? Please also provide dates.
   1. [Free text]
4. Are you of **black or minority ethnic (BME) background**?
   1. Yes
   2. No
5. (For participants who answered (i) Australia or (ii) New Zealand in Question 2) Are you of any of the following descents (select all applicable):
   1. Aboriginal
   2. Torres Strait Islander
   3. Māori
6. What **surgical specialty** are you undertaking training in?
   1. Cardiothoracic Surgery
   2. General Surgery
   3. Neurosurgery
   4. Oral and Maxillofacial Surgery
   5. Orthopaedic Surgery
   6. Otolaryngology, Head and Neck Surgery
   7. Paediatric Surgery
   8. Plastic and Reconstructive Surgery
   9. Urology
   10. Vascular Surgery
7. What **proportion of your expected operating activity** have you been able to perform this year?
   1. 0-100% (Visual Analogue Scale)
8. What **year of your surgical training** have you been undertaking in 2020?
   1. 1
   2. 2
   3. 3
   4. 4
   5. 5
   6. 6
   7. 7
   8. 8+
9. How many **surgical patients with a** **confirmed COVID-19 diagnosis** have you been directly involved in treating? Please provide a numerical estimate.
   1. [Numerical estimate]
10. Have **you or anyone in your family been diagnosed with COVID-19**?
    1. Myself
    2. Member of my family
    3. Neither
11. Did you **travel from overseas** to undertake your surgical training?
    1. Yes
    2. No
12. Since 11 March 2020, have you been undertaking your surgical training in a **full-time or less-than full-time fashion**?
    1. Full-time
    2. Less-than full-time
13. In the last month, how often have you felt that you were **unable to control the important things in your life**? [4-item Perceived Stress Scale]^18^
    1. Never (scored 0)
    2. Almost never (scored 1)
    3. Sometimes (scored 2)
    4. Fairly often (scored 3)
    5. Very often (scored 4)
14. In the last month, how often have you felt **confident about your ability to handle your personal problems**? [4-item Perceived Stress Scale]^18^
    1. Never (scored 4)
    2. Almost never (scored 3)
    3. Sometimes (scored 2)
    4. Fairly often (scored 1)
    5. Very often (scored 0)
15. In the last month, how often have you felt that **things were going your way**? [4-item Perceived Stress Scale]^18^
    1. Never (scored 4)
    2. Almost never (scored 3)
    3. Sometimes (scored 2)
    4. Fairly often (scored 1)
    5. Very often (scored 0)
16. In the last month, how often have you felt **difficulties were piling up so high that you could not overcome them**? [4-item Perceived Stress Scale]^18^
    1. Never (scored 0)
    2. Almost never (scored 1)
    3. Sometimes (scored 2)
    4. Fairly often (scored 3)
    5. Very often (scored 4)
17. **OHQ1:** How much do you agree or disagree with the following statement: **I don’t feel particularly pleased with the way I am** [8-item Oxford Happiness Questionnaire]^19^
    1. Strongly disagree (scored 6)
    2. Moderately disagree (scored 5)
    3. Slightly disagree (scored 4)
    4. Slightly agree (scored 3)
    5. Moderately agree (scored 2)
    6. Strongly agree (scored 1)
18. **OHQ2:** How much do you agree or disagree with the following statement: **I feel that life is very rewarding** [8-item Oxford Happiness Questionnaire]^19^
    1. Strongly disagree (scored 1)
    2. Moderately disagree (scored 2)
    3. Slightly disagree (scored 3)
    4. Slightly agree (scored 4)
    5. Moderately agree (scored 5)
    6. Strongly agree (scored 6)
19. **OHQ3:** How much do you agree or disagree with the following statement: **I am well satisfied about everything in my life** [8-item Oxford Happiness Questionnaire]^19^
    1. Strongly disagree (scored 1)
    2. Moderately disagree (scored 2)
    3. Slightly disagree (scored 3)
    4. Slightly agree (scored 4)
    5. Moderately agree (scored 5)
    6. Strongly agree (scored 6)
20. **OHQ4:** How much do you agree or disagree with the following statement: **I don’t think I look attractive** [8-item Oxford Happiness Questionnaire]^19^
    1. Strongly disagree (scored 6)
    2. Moderately disagree (scored 5)
    3. Slightly disagree (scored 4)
    4. Slightly agree (scored 3)
    5. Moderately agree (scored 2)
    6. Strongly agree (scored 1)
21. **OHQ5:** How much do you agree or disagree with the following statement: **I find beauty in some things** [8-item Oxford Happiness Questionnaire]^19^
    1. Strongly disagree (scored 1)
    2. Moderately disagree (scored 2)
    3. Slightly disagree (scored 3)
    4. Slightly agree (scored 4)
    5. Moderately agree (scored 5)
    6. Strongly agree (scored 6)
22. **OHQ6:** How much do you agree or disagree with the following statement: **I can fit in everything I want to** [8-item Oxford Happiness Questionnaire]^19^
    1. Strongly disagree (scored 1)
    2. Moderately disagree (scored 2)
    3. Slightly disagree (scored 3)
    4. Slightly agree (scored 4)
    5. Moderately agree (scored 5)
    6. Strongly agree (scored 6)
23. **OHQ7:** How much do you agree or disagree with the following statement: **I feel fully mentally alert** [8-item Oxford Happiness Questionnaire]^19^
    1. Strongly disagree (scored 1)
    2. Moderately disagree (scored 2)
    3. Slightly disagree (scored 3)
    4. Slightly agree (scored 4)
    5. Moderately agree (scored 5)
    6. Strongly agree (scored 6)
24. **OHQ8:** How much do you agree or disagree with the following statement: **I do not have particularly happy memories of the past** [8-item Oxford Happiness Questionnaire]^19^
    1. Strongly disagree (scored 6)
    2. Moderately disagree (scored 5)
    3. Slightly disagree (scored 4)
    4. Slightly agree (scored 3)
    5. Moderately agree (scored 2)
    6. Strongly agree (scored 1)
25. **PHQ1:** Over the last two weeks, how often have you been bothered by having **little interest or pleasure in doing things**? [PHQ-2 two-item depression screener]^28^
    1. Not at all (scored 0)
    2. Several days (scored 1)
    3. More than half the days (scored 2)
    4. Nearly everyday (scored 3)
26. **PHQ2:** Over the last two weeks, how often have you been bothered by **feeling down, depressed, or hopeless**? [Patient Health Questionnaire-2 (PHQ-2) two-item depression screener]^28^
    1. Not at all (scored 0)
    2. Several days (scored 1)
    3. More than half the days (scored 2)
    4. Nearly everyday (scored 3)
27. To what degree has a **loss of training opportunities, access to mandatory training courses or preferred rotations** associated with the COVID-19 pandemic affected your individual levels of stress?
    1. 0-10 rating scale
28. To what degree has **poor working conditions and ethical issues** associated with the COVID-19 pandemic affected your individual levels of stress?
    1. 0-10 rating scale
29. To what degree has **personal protective equipment (PPE) availability and your risk of exposure to COVID-19** affected your individual levels of stress?
    1. 0-10 rating scale
30. To what degree has the **relocation to areas of little expertise** associated with the COVID-19 pandemic affected your individual levels of stress?
    1. 0-10 rating scale
31. To what degree has the **difficulties with career progression and future job opportunities** associated with the COVID-19 pandemic affected your individual levels of stress?
    1. 0-10 rating scale
32. To what degree has the **loss of** **supervision and mentoring from consultants** associated with the COVID-19 pandemic affected your individual levels of stress?
    1. 0-10 rating scale
33. To what degree has the **difficulties with examinations, competence assessments, and interviews** associated with the COVID-19 pandemic affected your individual levels of stress?
    1. 0-10 rating scale
34. To what degree has the **difficulties with pay and contractual changes** associated with the COVID-19 pandemic affected your individual levels of stress?
    1. 0-10 rating scale
35. To what degree has the **difficulties obtaining leave and study leave** associated with the COVID-19 pandemic affected your individual levels of stress?
    1. 0-10 rating scale

***Table. Linear regression of Proportion expected operating activity versus seven day average daily COVID-19 case rates at location***

| Outcome | Predictor | Comparison | Mean difference (95% CI) | P value |
| --- | --- | --- | --- | --- |
| Proportion expected operating activity | Seven day average daily COVID-19 case rates at location | Per 1000 increase | -1.14 (-1.66, -0.63) | <0.0001 |

***Table. Multivariable linear regression of COVID-19 survey questions versus demographic variables***

| *Outcome* | *Predictor* | *Comparison* | *Mean difference (95%CI)* | *Comparison P value* | *Global P value* |
| --- | --- | --- | --- | --- | --- |
| A Loss training opportunities | Countries of training | Australia vs New Zealand | 2.79 (-7.68, 13.26) | 0.6019 | 0.0005 |
|  |  | Australia vs United Kingdom | -23.41 (-35.65, -11.17) | 0.0002 |  |
|  |  | New Zealand vs United Kingdom | -26.20 (-41.18, -11.22) | 0.0006 |  |
|  | Gender | Female vs Male | 11.72 (4.12, 19.32) |  | 0.0025 |
|  | Minority ethnic background | No vs Yes | -6.73 (-15.65, 2.18) |  | 0.1386 |
|  | Overseas travel | No vs Yes | 9.06 (-4.26, 22.38) |  | 0.1825 |
|  | Surgical Specialty | General vs Orthopaedic | -7.44 (-18.32, 3.43) | 0.1797 | 0.1960 |
|  |  | General vs Other | -6.60 (-15.08, 1.88) | 0.1269 |  |
|  |  | Orthopaedic vs Other | 0.84 (-11.00, 12.68) | 0.8889 |  |
|  | Year Surgical Training | 1 vs 2 | -3.47 (-14.55, 7.61) | 0.5397 | 0.0003 |
|  |  | 1 vs 3 | -4.81 (-16.60, 6.97) | 0.4234 |  |
| \|  \|  \| \| --- \| --- \| \|  \|  \| \|  \|  \| |  | 1 vs 4 | -18.03 (-29.71, -6.35) | 0.0025 |  |
|  |  | 1 vs 5+ | -22.20 (-33.17, -11.23) | <.0001 |  |
|  |  | 2 vs 3 | -1.35 (-14.49, 11.80) | 0.8410 |  |
|  |  | 2 vs 4 | -14.56 (-27.93, -1.20) | 0.0327 |  |
|  |  | 2 vs 5+ | -18.73 (-31.10, -6.36) | 0.0030 |  |
|  |  | 3 vs 4 | -13.22 (-26.96, 0.53) | 0.0594 |  |
|  |  | 3 vs 5+ | -17.39 (-30.43, -4.34) | 0.0090 |  |
|  |  | 4 vs 5+ | -4.17 (-17.24, 8.90) | 0.5319 |  |
| B poor working conditions | Countries of training | Australia vs New Zealand | 4.83 (-6.66, 16.33) | 0.4098 | 0.3509 |
|  |  | Australia vs United Kingdom | -9.63 (-26.53, 7.26) | 0.2639 |  |
|  |  | New Zealand vs United Kingdom | -14.47 (-34.10, 5.17) | 0.1487 |  |
|  | Estimate patients COVID |  | 2.52 (0.37, 4.67) |  | 0.0216 |
|  | Fulltime | Fulltime vs less than fulltime | 13.94 (-1.53, 29.40) |  | 0.0774 |
|  | Gender | Female vs Male | 10.18 (1.84, 18.53) |  | 0.0168 |
|  | Minority ethnic background | No vs Yes | -11.10 (-20.92, -1.28) |  | 0.0267 |
|  | Overseas travel training | No vs Yes | 14.01 (-0.40, 28.41) |  | 0.0567 |
|  | Year Surgical Training | 1 vs 2 | 0.09 (-11.98, 12.16) | 0.9881 | 0.0231 |
|  |  | 1 vs 3 | -0.83 (-13.70, 12.03) | 0.8991 |  |
|  |  | 1 vs 4 | -16.55 (-29.31, -3.79) | 0.0110 |  |
|  |  | 1 vs 5+ | -14.03 (-25.91, -2.15) | 0.0206 |  |
|  |  | 2 vs 3 | -0.92 (-15.34, 13.49) | 0.9000 |  |
|  |  | 2 vs 4 | -16.64 (-31.18, -2.10) | 0.0248 |  |
|  |  | 2 vs 5+ | -14.12 (-27.66, -0.59) | 0.0408 |  |
|  |  | 3 vs 4 | -15.72 (-30.77, -0.67) | 0.0407 |  |
|  |  | 3 vs 5+ | -13.20 (-27.50, 1.11) | 0.0705 |  |
|  |  | 4 vs 5+ | 2.52 (-11.55, 16.59) | 0.7256 |  |
| C personal protective equipment availability | Countries of training | Australia vs New Zealand | 5.55 (-5.54, 16.63) | 0.3267 | 0.6179 |
|  |  | Australia vs United Kingdom | 0.65 (-18.39, 19.69) | 0.9467 |  |
|  |  | New Zealand vs United Kingdom | -4.90 (-25.95, 16.16) | 0.6484 |  |
|  | Gender | Female vs Male | 19.72 (11.88, 27.57) |  | <.0001 |
|  | Overseas travel training | No vs Yes | 11.16 (-2.84, 25.16) |  | 0.1183 |
|  | Seven day COVID_19 |  | 0.11 (0.00, 0.21) |  | 0.0467 |
| D relocation areas little experience | Countries_trainingx | Australia vs New Zealand | 9.31 (-2.35, 20.96) | 0.1175 | 0.0625 |
|  |  | Australia vs United Kingdom | -13.47 (-30.90, 3.96) | 0.1299 |  |
|  |  | New Zealand vs United Kingdom | -22.78 (-42.30, -3.26) | 0.0222 |  |
|  | Diagnosed COVID19 | Family vs myself | 25.89 (-1.77, 53.56) | 0.0666 | 0.0490 |
|  |  | Family vs neither | 17.83 (2.05, 33.60) | 0.0267 |  |
|  |  | Myself vs Neither | -8.06 (-34.08, 17.95) | 0.5436 |  |
|  | Estimate patients COVID_19 |  | 1.56 (-0.47, 3.59) |  | 0.1310 |
|  | Gender | Female vs Male | 7.74 (-0.17, 15.66) |  | 0.0552 |
|  | Minority ethnic background | No vs Yes | -10.09 (-19.27, -0.92) |  | 0.0311 |
| E difficulties career progression | Countries_trainingx | Australia vs New Zealand | 6.94 (-5.93, 19.80) | 0.2908 | 0.0088 |
|  |  | Australia vs United Kingdom | -22.56 (-39.46, -5.65) | 0.0089 |  |
|  |  | New Zealand vs United Kingdom | -29.49 (-48.54, -10.45) | 0.0024 |  |
|  | Diagnosed_COVID19 | Family vs myself | 8.39 (-23.34, 40.11) | 0.6044 | 0.1068 |
|  |  | Family vs neither | 18.42 (1.24, 35.59) | 0.0356 |  |
|  |  | Myself vs Neither | 10.03 (-20.27, 40.33) | 0.5164 |  |
|  | Gender | Female vs Male | 6.46 (-2.51, 15.43) |  | 0.1582 |
|  | Overseas travel training | No vs Yes | 12.39 (-3.73, 28.51) |  | 0.1320 |
|  | Year Surgical Training | 1 vs 2 | -3.98 (-17.09, 9.12) | 0.5514 | <.0001 |
|  |  | 1 vs 3 | 0.05 (-13.69, 13.80) | 0.9940 |  |
|  |  | 1 vs 4 | -27.59 (-41.29, -13.90) | <.0001 |  |
|  |  | 1 vs 5+ | -36.82 (-49.58, -24.07) | <.0001 |  |
|  |  | 2 vs 3 | 4.04 (-11.69, 19.76) | 0.6150 |  |
|  |  | 2 vs 4 | -23.61 (-39.28, -7.94) | 0.0032 |  |
|  |  | 2 vs 5+ | -32.84 (-47.49, -18.19) | <.0001 |  |
|  |  | 3 vs 4 | -27.65 (-43.84, -11.45) | 0.0008 |  |
|  |  | 3 vs 5+ | -36.87 (-52.30, -21.45) | <.0001 |  |
|  |  | 4 vs 5+ | -9.23 (-24.47, 6.01) | 0.2353 |  |
| F loss supervision mentoring | Countries_trainingx | Australia vs New Zealand | 11.26 (-0.30, 22.82) | 0.0562 | 0.0382 |
|  | Countries_trainingx | Australia vs United Kingdom | -12.32 (-28.57, 3.92) | 0.1371 |  |
|  | Countries_trainingx | New Zealand vs United Kingdom | -23.59 (-42.50, -4.67) | 0.0145 |  |
|  | Estimate patients COVID-19 |  | 2.45 (0.31, 4.59) |  | 0.0248 |
|  | Gender | Female vs Male | 13.85 (5.33, 22.37) |  | 0.0014 |
|  | Overseas travel training | No vs Yes | 11.37 (-3.62, 26.37) |  | 0.1372 |
|  | Surgical Specialty | General vs Orthopaedic | -14.16 (-26.33, -1.98) | 0.0227 | 0.0649 |
|  |  | General vs Other | -0.51 (-9.93, 8.92) | 0.9162 |  |
|  |  | Orthopaedic vs Other | 13.65 (0.40, 26.90) | 0.0435 |  |
|  | Year Surgical Training | 1 vs 2 | 0.27 (-12.05, 12.59) | 0.9658 | 0.1292 |
|  |  | 1 vs 3 | 2.27 (-10.79, 15.32) | 0.7338 |  |
|  |  | 1 vs 4 | -3.51 (-16.43, 9.41) | 0.5945 |  |
|  |  | 1 vs 5+ | -14.21 (-26.48, -1.95) | 0.0231 |  |
|  |  | 2 vs 3 | 2.00 (-12.43, 16.42) | 0.7862 |  |
|  |  | 2 vs 4 | -3.78 (-18.36, 10.80) | 0.6116 |  |
|  |  | 2 vs 5+ | -14.48 (-28.18, -0.79) | 0.0382 |  |
|  |  | 3 vs 4 | -5.77 (-20.90, 9.36) | 0.4545 |  |
|  |  | 3 vs 5+ | -16.48 (-30.93, -2.02) | 0.0255 |  |
|  |  | 4 vs 5+ | -10.70 (-25.20, 3.79) | 0.1477 |  |
| G difficulties with examinations | Countries of training | Australia vs New Zealand | 3.19 (-10.00, 16.38) | 0.6355 | 0.0320 |
|  |  | Australia vs United Kingdom | -18.82 (-33.68, -3.95) | 0.0131 |  |
|  |  | New Zealand vs United Kingdom | -22.01 (-40.36, -3.66) | 0.0187 |  |
|  | Gender | Female vs Male | 11.82 (2.23, 21.42) |  | 0.0157 |
|  | Year Surgical Training | 1 vs 2 | 10.16 (-3.92, 24.24) | 0.1572 | <.0001 |
|  |  | 1 vs 3 | 2.49 (-12.14, 17.13) | 0.7384 |  |
|  |  | 1 vs 4 | -32.91 (-47.62, -18.21) | <.0001 |  |
|  |  | 1 vs 5+ | -26.23 (-39.87, -12.59) | 0.0002 |  |
|  |  | 2 vs 3 | -7.67 (-24.24, 8.90) | 0.3645 |  |
|  |  | 2 vs 4 | -43.07 (-60.03, -26.12) | <.0001 |  |
|  |  | 2 vs 5+ | -36.39 (-52.20, -20.57) | <.0001 |  |
|  |  | 3 vs 4 | -35.40 (-52.69, -18.12) | <.0001 |  |
|  |  | 3 vs 5+ | -28.72 (-44.98, -12.47) | 0.0005 |  |
|  |  | 4 vs 5+ | 6.68 (-9.71, 23.08) | 0.4243 |  |
| H difficulties with pay contractual | Countries of training | Australia vs New Zealand | 12.82 (1.40, 24.24) | 0.0277 | <.0001 |
|  |  | Australia vs United Kingdom | -30.28 (-45.03, -15.53) | <.0001 |  |
|  |  | New Zealand vs United Kingdom | -43.10 (-59.85, -26.35) | <.0001 |  |
|  | Diagnosed COVID19 | Family vs myself | 43.74 (16.07, 71.42) | 0.0019 | 0.0073 |
|  |  | Family vs neither | 4.82 (-10.17, 19.81) | 0.5285 |  |
|  |  | Myself vs Neither | -38.92 (-65.34, -12.51) | 0.0039 |  |
|  | Gender | Female vs Male | 6.47 (-1.44, 14.39) |  | 0.1090 |
|  | Overseas travel training | No vs Yes | 14.37 (0.31, 28.43) |  | 0.0452 |
|  | Year Surgical Training | 1 vs 2 | 10.56 (-1.11, 22.23) | 0.0761 | 0.0014 |
|  |  | 1 vs 3 | 6.99 (-5.20, 19.17) | 0.2610 |  |
|  |  | 1 vs 4 | -12.88 (-24.86, -0.89) | 0.0353 |  |
|  |  | 1 vs 5+ | -10.55 (-21.82, 0.72) | 0.0666 |  |
|  |  | 2 vs 3 | -3.58 (-17.61, 10.46) | 0.6175 |  |
|  |  | 2 vs 4 | -23.44 (-37.31, -9.57) | 0.0009 |  |
|  |  | 2 vs 5+ | -21.11 (-34.16, -8.07) | 0.0015 |  |
|  |  | 3 vs 4 | -19.86 (-34.11, -5.61) | 0.0063 |  |
|  |  | 3 vs 5+ | -17.54 (-31.19, -3.88) | 0.0118 |  |
|  |  | 4 vs 5+ | 2.33 (-11.04, 15.69) | 0.7330 |  |
| I difficulties obtaining leave | Countries of training | Australia vs New Zealand | 15.90 (3.88, 27.92) | 0.0095 | <.0001 |
|  |  | Australia vs United Kingdom | -32.13 (-45.74, -18.52) | <.0001 |  |
|  |  | New Zealand vs United Kingdom | -48.03 (-64.76, -31.31) | <.0001 |  |
|  | Fulltime | Fulltime vs less than fulltime | 11.06 (-5.27, 27.39) |  | 0.1844 |
|  | Gender | Female vs Male | 11.74 (2.95, 20.53) |  | 0.0089 |
|  | Overseas travel training | No vs Yes | 11.18 (-3.99, 26.35) |  | 0.1487 |
|  | Year Surgical Training | 1 vs 2 | 4.54 (-8.02, 17.10) | 0.4789 | 0.0553 |
|  |  | 1 vs 3 | 8.44 (-5.27, 22.15) | 0.2275 |  |
|  |  | 1 vs 4 | -13.58 (-27.12, -0.03) | 0.0494 |  |
|  |  | 1 vs 5+ | -5.99 (-18.60, 6.62) | 0.3521 |  |
|  |  | 2 vs 3 | 3.90 (-11.33, 19.14) | 0.6156 |  |
|  |  | 2 vs 4 | -18.12 (-33.45, -2.78) | 0.0206 |  |
|  |  | 2 vs 5+ | -10.52 (-24.74, 3.69) | 0.1467 |  |
|  |  | 3 vs 4 | -22.02 (-38.25, -5.79) | 0.0078 |  |
|  |  | 3 vs 5+ | -14.43 (-29.82, 0.96) | 0.0661 |  |
|  |  | 4 vs 5+ | 7.59 (-7.54, 22.72) | 0.3255 |  |

**Figure. First half of primary hypothesis – Outcome: PSS Total Score, predictors: demographic and COVID-19 - Checking the assumptions of a linear model**

**Figure. Part 2 primary hypothesis – Outcome: PSS Total Score, predictor: Seven_day_average_daily_COVID-19 - Checking assumptions of a linear regression**

**Figure. Outcome: OHQ Total Score, predictors: demographic and COVID-19 - Check assumption of linear regression**

**Figure. Outcome: OHQ Total Score, predictor: Seven_day_average_daily_COVID-19 - Checking assumptions of a linear regression**

**Figure. Outcome: PHQ Total Score, predictors: demographic and COVID-19 - Check assumption of linear regression**

**Figure. Outcome: PHQ Total Score, predictor: Seven_day_average_daily_COVID-19 - Checking assumptions of a linear regression**

**Figure. Outcome: Expected operating activity, predictors: demographic and COVID-19 - Check assumption of linear regression**

**Figure. Outcome: Expected operating activity, predictor: Seven_day_average_daily_COVID-19 - Checking assumptions of a linear regression**
